# The relationship between monoaminergic gene expression, learning, and optimism in red junglefowl chicks

**DOI:** 10.1007/s10071-020-01394-z

**Published:** 2020-05-21

**Authors:** Robert Boddington, Clara A. Gómez Dunlop, Laura C. Garnham, Sara Ryding, Robin N. Abbey-Lee, Anastasia Kreshchenko, Hanne Løvlie

**Affiliations:** 1 Department of Physics, Chemistry and Biology, IFM Biology, Linköping University, 581 83 Linköping, Sweden; 2 School of Biological Sciences, University of Manchester, Manchester, M13 9PL UK

**Keywords:** Animal cognition, Associative learning, Cognitive flexibility, Dopamine, *Gallus gallus*, Judgement bias, Serotonin

## Abstract

Intra-species cognitive variation is commonly observed, but explanations for why individuals within a species differ in cognition are still understudied and not yet clear. Cognitive processes are likely influenced by genetic differences, with genes in the monoaminergic systems predicted to be important. To explore the potential role of these genes in association with individual variation in cognition, we exposed red junglefowl (*Gallus gallus*) chicks to behavioural assays measuring variation in learning (discriminative learning, reversal learning, and cognitive flexibility) and optimism (measured in a cognitive judgement bias test). Following this, we analysed prefrontal cortex gene expression of several dopaminergic and serotonergic genes in these chicks. Of our explored genes, serotonin receptor genes 5HT2A and 5HT2B, and dopaminergic receptor gene DRD1 were associated with measured behaviour. Chicks that had higher 5HT2A were less flexible in the reversal learning task, and chicks with higher 5HT2B also tended to be less cognitively flexible. Additionally, chicks with higher DRD1 were more optimistic, whilst chicks with higher 5HT2A tended to be less optimistic. These results suggest that the serotonergic and dopaminergic systems are linked to observed cognitive variation, and, thus, individual differences in cognition can be partially explained by variation in brain gene expression.

**Electronic supplementary material:**

The online version of this article (10.1007/s10071-020-01394-z) contains supplementary material, which is available to authorized users.

## Introduction

Intra-species variation in animal cognition (i.e., how animals perceive, process, retain, and act on cues from their environment, Shettleworth 2010) is commonly observed (Dukas 2004; Thornton and Lukas 2012) and can have fitness consequences (Dukas 2004; Shaw et al. 2019). Nevertheless, the underlying mechanisms behind this variation, such as the role of genes, remain largely unknown (Dukas 2004; Croston et al 2015).

Research on cognitive variation typically focuses on associative learning (i.e., learning predictable relationships between events, Shettleworth 2010), a key aspect of cognition. Associative learning includes discriminative learning (i.e., learning to perform different responses to two or more stimuli) and reversal learning (i.e., the process in which a previously learnt association is extinguished and a new one formed, Shettleworth 2010). Cognitive flexibility can be measured through associative learning tasks, for example, in reversal learning, where animals need to learn new information, whilst retaining or forgetting older information (e.g., Strang and Sherry 2014; Shettleworth 2010). This flexibility is evolutionarily important, as it enables animals to respond to ever-changing environments and situations, allowing them to adapt to and overcome challenges as they arise (Morand-Ferron 2017). Despite the importance of such behavioural and cognitive flexibility, within-species variation still occurs. A proactive–reactive personality gradient can explain some of this variation, where proactive individuals are generally less flexible than their reactive conspecifics (Koolhaas et al. 1999; Coppens et al. 2010). Both discriminative and reversal learning performance can be heritable (e.g., insects, *Apis mellifera*, Ferguson et al. 2001; *Drosophila melanogaster*, Kawecki 2010; mice, *Mus musculus*, Laughlin et al. 2011; rats, *Rattus norvegicus*, Shumake et al. 2014), suggesting that genetic differences between individuals may underlie variation in both performance in learning tasks and cognitive flexibility. Nevertheless, it is not clear which genes underlie this variation.

Cognitive processes can contain biases (Fawcett et al. 2013), for example, when variation in the affective state produces optimistic or pessimistic biases (Mendl et al. 2009). A positive affective state results in unfamiliar ambiguous stimuli, intermediate of stimuli with known values (i.e., judgement bias test, Harding et al. 2004), being interpreted more optimistically (e.g., dogs, *Canis lupis familiaris*, Burman et al. 2011; rats, Brydges et al. 2011; Rygula et al. 2012; domestic chickens, *Gallus domesticus*, Zidar et al. 2018b). Such optimistic bias can be influenced by selection, demonstrating a genetic contribution (Fawcett et al. 2013).

The dopaminergic and serotonergic systems play fundamental roles in explaining behavioural variation (Winberg and Nilsson 1993; Swallow et al. 2016), and so could be expected to underlie variation in learning, cognitive flexibility, and optimism. The dopaminergic system is involved in establishing and strengthening associations between stimulus and reward (Shultz et al. 1997; Frank et al. 2004). This is supported by the firing strength of dopaminergic neurons and dopamine release, increasing during discriminative learning tasks (rats, Stuber et al. 2008; gerbils, *Meriones unguiculatus*, Stark et al. 2004). Specifically, dopamine receptors D1 and D2 are linked to variation in such tasks (e.g., Beninger and Miller 1998; Puig et al. 2014). Reversal learning performance is also affected by the dopaminergic system (reviewed in Kehagia et al. 2010). Both depleted dopamine levels and selective D2 receptor antagonists can impair reversal learning (e.g., humans, Mehta et al. 2004; marmoset monkey, *Callithrix jacchus*, Walker et al. 2008; rats, Floresco et al. 2006; vervet monkeys, *Chlorocebus aethiops sabaeus,* Lee et al. 2007; mice, DeSteno and Schmauss 2009). Taken together, this suggests that variation in dopaminergic genes, specifically DRD1 and DRD2, could underlie variation in associative learning.

The role of the serotonergic system in associative learning is currently not well understood. Whilst levels of serotonin are believed to be independent of learning and memory processes (Bacqué-Cazenave et al. 2020), serotonergic receptors and tryptophan hydroxylase (TPH, an enzyme essential for the synthesis of serotonin from tryptophan), are implicated specifically in associative learning (Harvey 2003; Izquierdo et al. 2012; Bacqué-Cazenave et al. 2020). For example, inhibiting or knocking out TPH genes, as well as 5HT1A antagonism, impairs associative learning performance (rats, Izquierdo et al. 2012; nematodes, *Caenorhabditis elegans,* Nuttley et al. 2002; fish, *Labroides dimidiatus*, Soares et al. 2016). For discriminative learning, specifically, the role of serotonin is unclear. Some studies conclude that serotonin depletion facilitates discriminative learning (Graham et al. 1994; Ward et al. 1999), whilst other studies conclude the opposite (Harrison et al. 1999; Iigaya et al. 2018). In reversal learning, a lack of serotonin, either through brain damage or lack of tryptophan, impairs performance (e.g., marmoset monkey, Clarke et al. 2004; humans, Park et al. 1994) potentially because serotonin is needed for inhibiting previously learnt responses (Clarke et al. 2007). Similarly, serotonin has been implicated in modulating cognitive flexibility (Clarke et al. 2007). Cognitive flexibility is sometimes linked to the proactive–reactive personality gradient, where decreased serotonergic input may be linked to the lower flexibility exhibited by proactive individuals (Coppens et al. 2010). The role of genetic variation in serotonergic genes in reversal learning is not well understood. Selective 5HT2A receptor antagonists can both improve (mice, Baker et al. 2011; Amodeo et al. 2014) and impair reversal learning (rats, Boulougouris et al. 2008). 5HT2C receptor antagonism, thus far, appears to improve reversal learning (rats, Boulougouris et al. 2008; mice, Nilsson et al. 2012), but whether this is true outside of rodents is unclear.

Changes in the dopaminergic and serotonergic system may result in more optimistic or pessimistic judgement biases (Sharot et al. 2012; Anderson et al. 2013; Neville et al. 2020). Elevated brain levels of dopamine are linked with increased optimism (humans, Sharot et al. 2012; domestic chickens, Zidar et al. 2018b), whilst lowered levels are linked with increased pessimism (honeybees, *Apis mellifera carnica*, Bateson et al. 2011; bumblebees, *Bombus terrestris*, Perry et al. 2016). Polymorphisms in D2 receptor genes have been associated with avoidance-based decisions, similar to a pessimistic bias (Frank and Hutchison 2009), therefore, indicating a more specific receptor-based link between dopamine and processing emotionally relevant stimuli (Blasi et al. 2009). The role of the D1 receptor in optimism bias, to our knowledge, has not yet been considered. How serotonin affects judgement bias is less clear. Thus far, inhibiting TPH causes a pessimistic bias towards ambiguous stimuli (sheep, *Ovis aries*, Doyle et al. 2011) and 5HT2A receptors have been suggested to be involved in emotion-based decision-making (Aznar and Klein 2013), which can be linked to judgement biases (Gibson and Sanbonmatsu 2004). Predictions are currently lacking with regards to how other serotonergic receptors may affect optimism. Pharmacological manipulations overall altered judgement biases as predicted (reviewed in Neville et al. 2020), but not many studies have yet investigated the effects of manipulating monoaminergic systems on judgement bias, and these produced inconclusive results (Rygula et al. 2014; Golebiowska and Rygula, 2017). Overall, more research is needed to clarify the role of different dopaminergic and serotonergic genes in optimism.

We here explore if variation in brain gene expression of genes of the dopaminergic (DRD1 and DRD2) and serotonergic systems (TPH, 5HT1B, 5HT2A, 5HT2B, and 5HT2C) influences variation in discriminative learning, reversal learning, cognitive flexibility, and optimism in red junglefowl, *Gallus gallus.* Red junglefowl are commonly used in behavioural and cognition research (reviewed by Garnham and Løvlie 2018). Genes DRD1, DRD2, TPH, 5HT2A, and 5HT2C were chosen, because earlier studies implicate variation in these genes in the variation of cognitive traits (Ryding et al. unpublished; Boulougouris et al. 2008). Genes 5HT1B and 5HT2B were chosen as, while they have not yet been investigated in the context of associative learning and optimism, they can play a role in other aspects of cognition such as memory and inhibitory control (Buhot et al. 1995; Tikkanen et al. 2015). Based on previous work across a range of species, we predicted that discriminative learning performance would be positively correlated with gene expression of dopamine receptor expression, whilst reversal learning performance and optimism would be positively correlated with both dopamine and serotonin receptor gene expression. We hypothesised that cognitive flexibility would also be positively correlated with serotonin receptor gene expression. We additionally predicted that reversal learning performance would be positively correlated with TPH gene expression.

## Methods

### Animals and housing

We used 33 red junglefowl from a pedigree bred population at Linköping University (see Sorato et al. 2018 for further details on this population). Chicks were hatched in artificial incubators (to reduce maternal effects), and wing-tagged with unique numbers. For the duration of the study, chicks were housed in mixed-sex groups (≤ 25 individuals) together with non-test birds in cages (72 × 71 × 53 cm, L × W × H) equipped with perches, heaters, light (7 am–7 pm), and with ad libitum commercial poultry feed and water*.* After 5 weeks of age, chicks were moved to a chicken facility outside of Linköping (for more information, see Zidar et al. 2018a). Chicks were sexed at 6 weeks of age, when moulted into sex-specific plumage, and thus, sex was unknown until after behavioural testing was finished. The experiment was carried out in accordance with Swedish ethical requirements (Linköping Ethical Committee, ethical permit numbers 50-13).

### Experimental set-up

To reduce learning impairment due to stress, we habituated chicks to being alone in the test arena by gradually reducing the number of individuals in the test arena (72 × 71 × 53 cm, *L* × *W* × *H*), while feeding them pieces of mealworms, until they showed no signs of isolation stress (sensu Zidar et al. 2017b). After habituation to the set-up, all chicks singly took part in discriminative learning tasks at 3–4 days old, reversal learning tasks at 5–6 days old, and cognitive judgement bias tests at 12–13 days old. At 14–19 days of age, chicks took part in a detour-reaching test to measure impulsivity (as part of another study, Ryding et al. unpublished). Chicks were given ≥ 1 h of rest in their home pen before testing continued between sessions, to maintain their motivation. All testing took place between 8 am and 6 pm (local time), and all chicks (*n* = 33, *n*_males_ = 19, *n*_females_ = 14) that took part in these tests passed them; however, some chicks did not participate in all the tests (for unbiased, logistical reasons).

### Discriminative learning

In our discriminative learning task (sensu Zidar et al. 2017b; Sorato et al. 2018), each chick (*n* = 33) had to learn to associate a black stimulus (a bowl: 5 × 3 cm, *Ø* × *H*, with a 9 cm^2^ card behind it) with a reward (a piece of mealworm), and a white stimulus (same sized bowl and card) with no reward. We presented these stimuli simultaneously, and chicks passed this task when they chose the rewarded stimulus (by approaching with their head within 2 cm of it), in six consecutive presentations. Learning performance was measured as the number of trials needed to reach this criterion (termed ‘discriminative learning performance’). We gave each chick up to seven sessions (a session ended when 30 stimuli presentations had been made, or ca 15 min had elapsed, whichever came first) to pass this task. In the initial trials, the experimenter would guide chicks toward the stimuli, but guiding stopped as soon as a chick would actively explore these on its own.

### Reversal learning

In the reversal learning task (sensu Zidar et al. 2017b; Sorato et al. 2018), we presented each chick (*n* = 33) with the same stimuli as in the discriminative learning task, but now the white stimulus was rewarded, and the black was not. Each chick was given 5 min to stop inspecting the black stimulus (unrewarded) and approach the white (now rewarded) stimulus during their first presentation. The latency to stop choosing the previously rewarded stimulus was recorded in seconds (‘reversal learning latency’). This was used as a measure of behavioural and cognitive flexibility (Zidar et al. 2017a; Zidar et al. 2019). Each chick was given 3 of these 5 min sessions to approach the white stimulus. If the chick did not approach after these sessions they were taught the new discrimination by the observer showing them the reward in the white bowl. Subsequent presentations did not include this opportunity to receive help, and instead used the same criteria as for discriminative learning regarding initial choice made, number of stimuli presentations in a session, number of sessions, and criteria for passing this task (‘reversal learning performance’).

### Cognitive judgement bias test

In a judgement bias test (sensu Zidar et al. 2018b), chicks (*n* = 30) were presented with one colour stimuli (a bowl and a card) at the time. Colour stimuli were the original white and black stimuli used in our learning tasks, plus an additional 3 grey stimuli, which were intermediate between the stimuli used in the learning tasks and, thus, ambiguous in signal (‘light grey’ 75%white/25%black; ‘mid grey’: 50%white/50%black; ‘dark grey’: 25%white/75%black). Only the white stimulus was rewarded. Chicks were subject to 30 stimuli presentations with a maximum of 30 s given for each presentation. Latency (in seconds) to approach each stimulus (within 2 cm of it) was recorded. A shorter latency to approach ambiguous cues indicates higher optimism and a more positive affective state (Mendl et al. 2009; Sorato et al. 2018; Garnham et al. 2019). Individuals that did not approach within 30 s were given 30 s as latency. In the current study, we only used average latency to mid-grey to measure optimism (the other grey cues in this test were used as part of another study investigating the heritability of cognitive traits, including optimism, in our population, Sorato et al. 2018). Our previous work has shown that response to the mid-grey cues typically strongly correlates within individuals with responses to the other ambiguous, grey cues (Zidar et al. 2018b; Garnham et al. 2019).

### Gene expression analysis

To examine the relationship between gene expression of serotonergic and dopaminergic genes and our cognitive measures, we culled the chicks (*n* = 33) at 9 weeks old by rapid decapitation and dissected their brains for gene expression analysis. The caudal region of the left telencephalon was extracted and snap-frozen in liquid nitrogen (≤ 4 min) and stored at − 80 °C until RNA extraction. We chose these areas as the left hemisphere is the dominant hemisphere for cognition, such as the control of motivational and emotional responses (Vallortigara et al. 1999), and the prefrontal cortex including the caudal region, in particular, is implicated in learning and optimism (Aznar and Klein 2013; Puig et al. 2014).

RNA was extracted using Ambion TRI Reagent (Life Technologies, USA) according to the manufacturer's instructions. The extracted RNA of all samples was measured using Nanodrop 1000 (Thermofisher, Sweden), and the quality of RNA measured using Agilent 2100 Bioanalyzer for a subset of 12 individuals. All RNA integrity number values were above 9, showing that the samples were not degraded. Single-stranded cDNA was prepared using Maxima First Strand cDNA Synthesis Kit with DNase (Thermo Fisher Scientific, USA) using 1 µg total RNA as a template. The primers used targeted POL2 and TBP for housekeeper genes, dopamine receptor genes DRD1 and DRD2, and serotonin receptor genes 5HT1B, 5HT2A, 5HT2B, and 5HT2C, and serotonin synthesiser TPH (supplementary Table S1)*.* Primer specificity was ensured by examination of the melting curve run on pooled cDNA from all individuals. Each 10 µl reaction volume used for the qPCR contained 1 µl of equal parts forward and reverse primer, 60–80 picograms of cDNA diluted in 2 µl water, 5 µl SYBR Green I Master (Roche Diagnostics, Switzerland), and 2 µl water. The qPCR was performed in a Light Cycler 480 (Roche Diagnostics, Switzerland) at 5 min 95 °C for activation, succeeded by 40 cycles (10 s 95 °C, 10 s 60 °C, and 20 s 72 °C). The end of the program ran a melting curve from 72 to 95 °C, before cooling to 40 °C.

One of the plates (plate 1 of 22) was eliminated from analysis due to a calibration error during PCR. We calculated the crossing point (*C*_p_) values over the two housekeeping genes. Because samples were run in duplicate, the average expression was calculated for each individual based on the two *C*_p_ values (CV = 18.07%) The expression levels of the genes of interest were calculated for each individual by the difference in expression between the housekeeper genes and the gene of interest (Δ*C*_p_). Higher Δ*C*_p_ values signify lower gene expression.

### Statistical analyses

R version 1.2.1335 (R Core team 2019) was used for statistical analyses.

Our cognitive measures (discriminative learning performance, reversal learning performance, reversal learning latency, and optimism) did not follow the assumptions for parametric statistics, so we used non-parametric statistics to analyse these. To investigate the effects of sex on cognitive measures (e.g., Vallortigara 1990; Favati et al 2016; Zidar et al. 2018a) and gene expression levels, we used Mann–Whitney *U* tests. To explore correlations among cognitive measures, and between cognitive measures and gene expression levels, we used Spearman rank correlations. As some chicks were not tested in all cognitive tasks, sample sizes vary somewhat between comparisons. Due to the presence of some extreme values in 5HT2A expression in our comparisons (see results), analyses were run with and without these values, which did not affect the direction of observed relationships (Supplementary Information, Figure S1–S3). Results including these values are presented here.

## Results

### Effect of sex

The sexes did not differ in cognitive measures or gene expression levels (*W* > 147.00, *p* > 0.10), except for DRD1 (*W* = 85.50, *p* = 0.087). Therefore, the relationship for each sex for DRD1 expression and each behavioural measure were visually inspected. The direction of these relationships was in a similar direction for both sexes, and data from both sexes were, therefore, pooled for further analyses.

### Correlation between cognitive measures

There was a negative correlation between reversal learning performance and cognitive flexibility (i.e., reversal learning latency, *n* = 31, *r*_s_ = − 0.51, *p* = 0.003). Other cognitive measures did not correlate (*n* = 30–33, *r*_s_ ≤  ± 0.20, *p* ≥ 0.25).

### Cognitive measures and brain gene expression

**Fig. 1:**
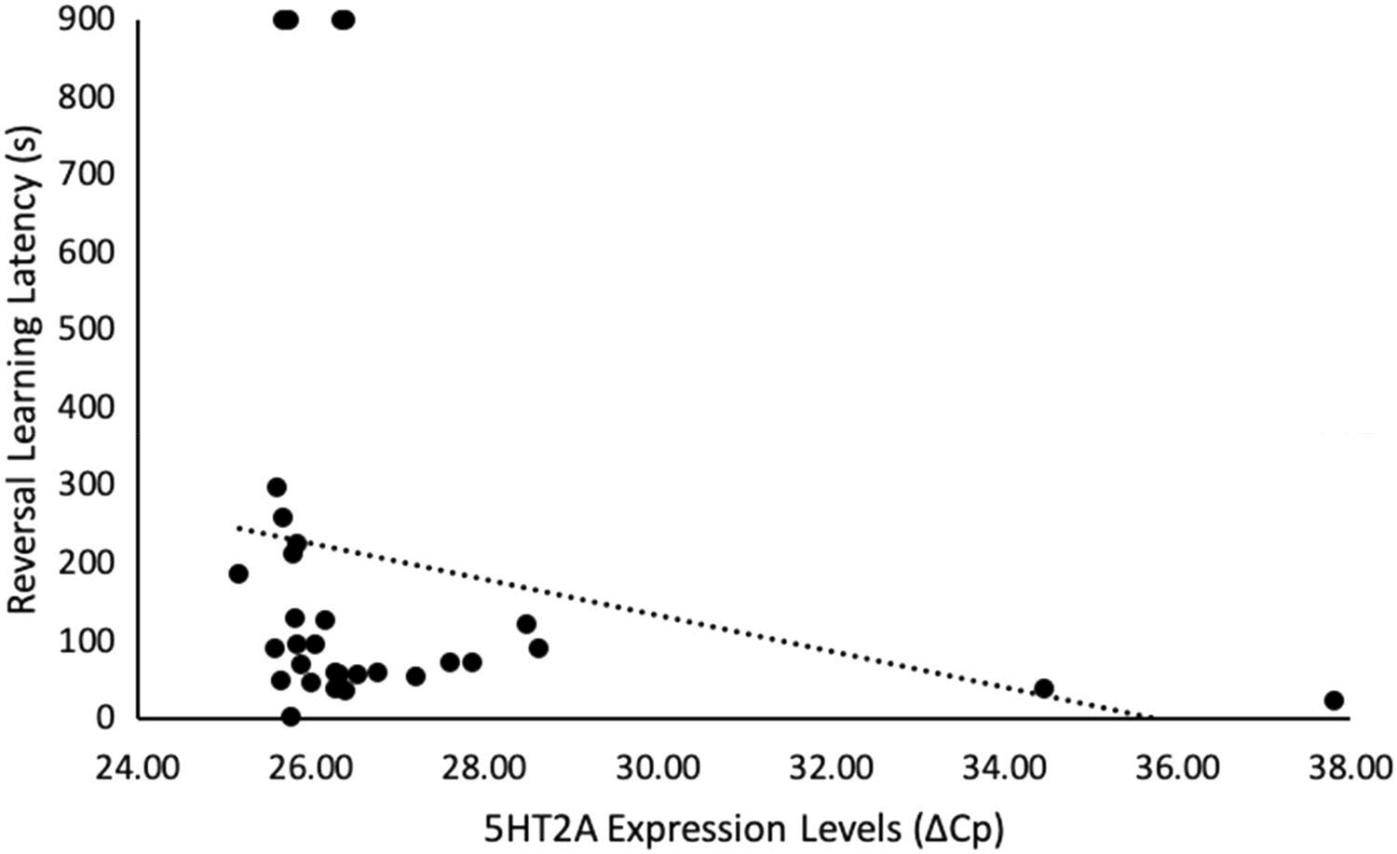
Relationship between reversal learning latency and 5HT2A gene expression levels in red junglefowl chicks. ‘Reversal learning latency’ is latency (in seconds) to stop choosing a previously rewarded stimulus in a reversal learning task and instead choose the now rewarded stimulus. A longer latency indicates a less flexible response. Gene expression levels are measured by ΔCp, which is the difference between the gene of interest and a housekeeper gene. Higher ΔCp value indicates lower expression levels. Re-analyses of the relationship after removal of the two extreme gene expressions, or removal of also the four extreme reversal latency values, did not alter the direction of observed relationship (Supplementary Information, Fig. S1a and b, respectively)

**Fig. 2:**
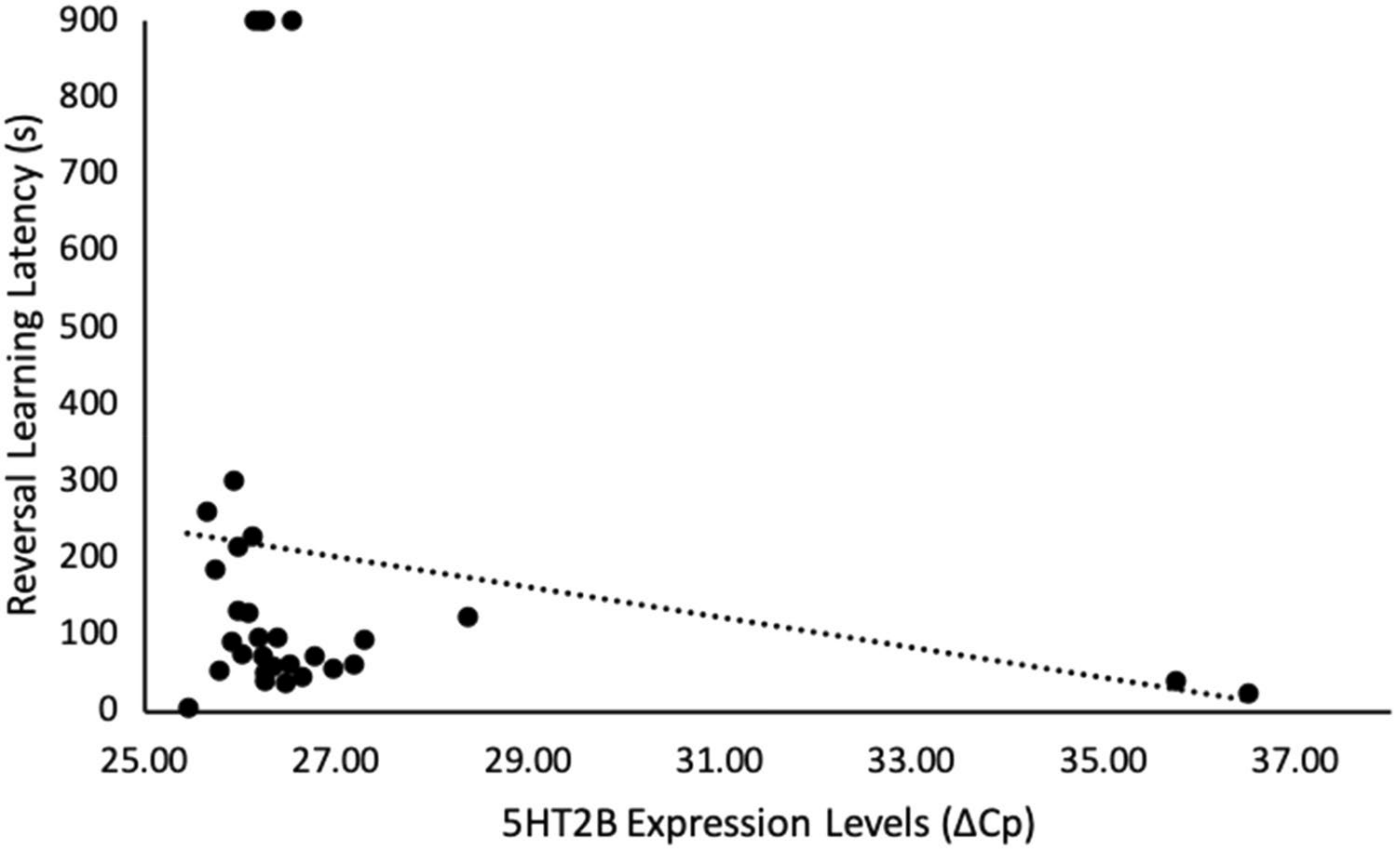
Relationship between reversal learning latency and 5HT2B gene expression levels in red junglefowl chicks. ‘Reversal learning latency’ is latency (in seconds) to stop choosing a previously rewarded stimulus in a reversal learning task and instead choose the now rewarded stimulus. A longer latency indicates a less flexible response. Gene expression levels are measured by ΔCp, which is the difference between the gene of interest and a housekeeper gene. Higher ΔCp value indicates lower expression levels. Re-analyses of the relationship after removal of the two extreme gene expressions, and also the four extreme reversal latency values did not alter the direction of observed relationship (Supplementary Information, Fig. S2a and b, respectively)

**Fig. 3:**
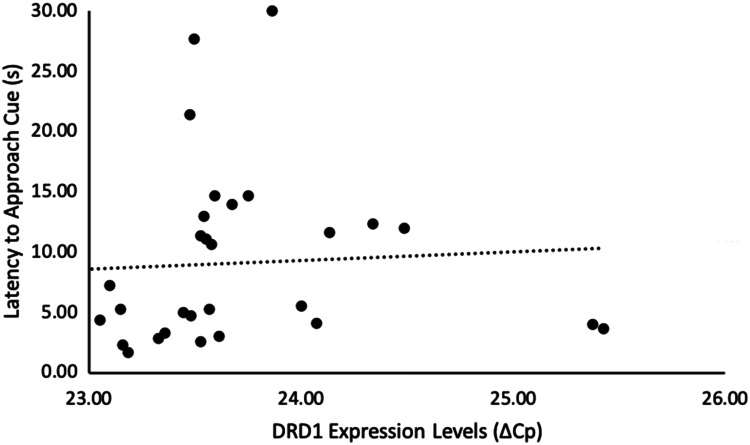
Relationship between optimism and DRD1 gene expression levels in red junglefowl chicks. ‘Latency to approach cue’ is the used measure of optimism, which is measured as latency (in seconds) to approach a novel, intermediate stimulus in a judgement bias test. A shorter latency indicates a more optimistic response. Gene expression levels are measured by ΔCp, which is the difference between the gene of interest and a housekeeper gene. Higher ΔCp value indicates lower expression levels

**Fig. 4:**
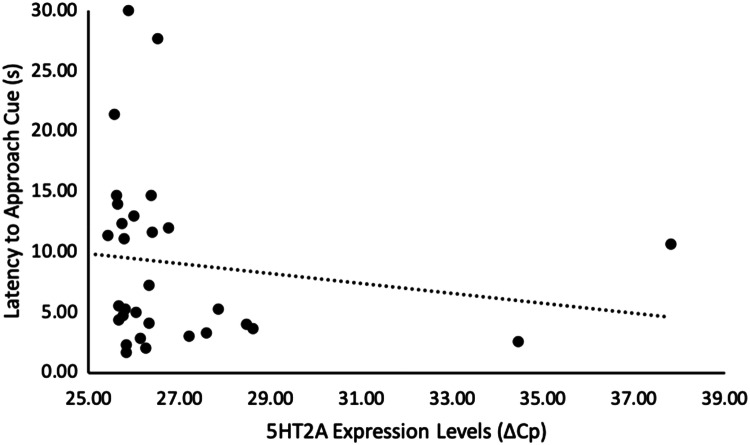
Relationship between optimism and 5HT2A gene expression levels in red junglefowl chicks. ‘Latency to approach cue’ is the used measure of optimism, which is measured as latency (in seconds) to approach a novel, intermediate stimulus in a judgement bias test. A shorter latency indicates a more optimistic response. Gene expression levels are measured by ΔCp, which is the difference between the gene of interest and a housekeeper gene. Higher ΔCp value indicates lower expression levels. Re-analyses of the relationship after removal of the two extreme gene expression values (to the right), did not alter the direction of observed relationship (Supplementary Information, Fig. S3)

Of the genes we examined, genes of both the dopamine (DRD1) and serotonin (5HT2A and 5HT2B) systems correlated with our cognitive measures. Chicks that were less cognitively flexible (i.e., slower to approach the formerly unrewarded, but now rewarded, stimulus in a reversal learning task) had higher levels of 5HT2A (*n* = 31, *r*_s_ = − 0.39, *p* = 0.029, Fig. 1, Table 1), and tended to have higher levels of 5HT2B gene expression (*n* = 31, *r*_s_ = − 0.33 *p* = 0.074, Fig. 2, Table 1). Additionally, DRD1 expression was higher, and 5HT2A expression was lower, in chicks that were more optimistic (i.e., had shorter latencies to approach the ambiguous novel stimulus in a cognitive judgement bias test) (DRD1: *n *= 30, *r*_s_ = 0.36, *p* = 0.048, Fig. 3, Table 1; 5HT2A: *n* = 30, *r*_s_ = − 0.31, *p* = 0.095, Fig. 4, Table 1). None of our other genes investigated associated with any other of our cognitive measures taken (*n* = 30–33, *r*_s_ < 0.30, *p* > 0.10, Table 1).

**Table 1:** Relationships between cognitive measures and brain gene expression, in red junglefowl chicks

Gene expression	Discriminative learning performance	Reversal learning performance	Reversal learning latency	Optimism
	*R*_s_ (*p*)	*R*_s_ (*p*)	*R*_s_ (*p*)	*R*_s_ (*p*)
DRD1	− 0.058 (0.75)	− 0.0071 (0.97)	− 0.018 (0.93)	**0.36 (0.048)**
DRD2	− 0.11 (0.55)	0.25 (0.16)	− 0.28 (0.13)	− 0.17 (0.38)
5HT1B	− 0.15 (0.42)	0.26 (0.14)	0.046 (0.81)	0.19 (0.32)
5HT2A	0.28 (0.11)	0.24 (0.18)	− **0.39 (0.029)**	− *0.31 (0.095)*
5HT2B	0.18 (0.31)	0.21 (0.23)	− *0.33 (0.074)*	− 0.019 (0.92)
5HT2C	0.061 (0.73)	0.28 (0.11)	− 0.091 (0.63)	0.066 (0.73)
TPH	0.098 (0.59)	0.25 (0.16)	− 0.19 (0.32)	0.069 (0.72)

‘Discriminative learning performance’ is the number of trials needed until a discriminative learning task was learnt (*n* = 33), ‘Reversal learning performance’ is the number of trials needed until a reversal learning task was learnt (*n* = 33), ‘Reversal learning latency’ is latency (in seconds) to stop choosing a previously rewarded stimulus in a reversal learning task and instead choose the now rewarded stimulus (*n* = 31), ‘Optimism’ was measured as latency to approach a novel, intermediate stimulus in a judgement bias test (*n* = 30). ‘DRD1’ and ‘DRD2’ are dopaminergic receptors, ‘5HT1B’, ‘5HT2A’, ‘5HT2B’, and ‘5HT2C’ are serotonergic receptors, and ‘TPH’ is a serotonin synthesiser. Spearman correlation coefficient (*R*_s_) and corresponding *p* value (*p*) are given. Bold = *p* < 0.05, italic = *p* <  0.10

## Discussion

We here explored the relationship between within-species variation in aspects of cognition in red junglefowl chicks, focusing on two aspects of learning (discriminative and reversal learning), cognitive flexibility, optimism, and brain gene expression of genes from two monoaminergic systems (dopamine, serotonin). We found that chicks with higher 5HT2A brain gene expression were less flexible in a reversal learning task (i.e., were slower to approach the new, rewarded stimulus). A similar pattern tended to emerge for chicks that had higher 5HT2B expression. Furthermore, chicks that had higher DRD1 expression were more optimistic, whereas chicks with higher 5HT2A expression tended to be less optimistic.

Dopaminergic receptors have been implicated in associative learning, specifically D1 and D2 receptors in discriminative learning (e.g., Puig et al. 2014), and D2 receptors in reversal learning (e.g., Lee et al. 2007). Contrary to these findings, and our initial hypotheses, our study found no association between these receptor genes with the facets of associative learning here explored. The previous studies have mostly focused on mammals, thus, further work is needed to elucidate the role of the dopaminergic system in birds and determine how this differs from patterns found in mammals.

The serotonergic system is suggested to play a role in cognitive flexibility (Clarke et al. 2007) and can influence variation along a proactive–reactive gradient (Coppens et al. 2010; Koolhaas et al. 2007). Proactive individuals are typically less flexible and tend to rely more on routines (Coppens et al. 2010). These individuals have been described to typically perform worse when attempting to extinguish previously reinforced responses in a reversal learning task (pigs, Bolhuis et al. 2004). This response is very similar to the high reversal learning latency that we observed in chicks with high 5HT2A and 5HT2B expression who appeared to be less flexible and have difficulty extinguishing the previously reinforced response. Previous work in our population found that reversal learning latency correlated with inflexibility in responses to the configuration of a spatial task (Zidar et al 2017a), and fearfulness (Zidar et al 2019). Our results, therefore, suggest that 5HT2A and 5HT2B expression levels play a role in explaining individual variation in proactivity and cognitive flexibility. Furthermore, as we analysed the gene expression in the prefrontal cortex, which has been implicated in both mammalian and avian flexibility and choice behaviour (Dalley et al. 2008; Matsushima et al. 2008), our results may suggest that these receptors in this brain region are the ones contributing to the variation in flexibility observed. This warrants further investigation to confirm.

The general role of serotonin in associative learning has been established (Harvey 2003). While different studies contrast each other in terms of its role in discriminative learning (Harrison et al. 1999; Graham et al. 1994; Ward et al. 1999), its role in reversal learning is clearer, with increased serotonin levels improving performance in this task (Clarke et al. 2007). However, the absence of associations between the serotonergic system and the facets of associative learning which we examined may suggest that our chosen receptors do not play a role in associative learning in birds, at least not in red junglefowl. As a majority of cognitive studies are conducted in mammals, these findings suggest a need for more research into causal mechanisms of non-mammalian cognition. Moreover, we did not find any direct association between discriminative and reversal learning. This lack of a phenotypic correlation between these aspects of learning confirms the previous work on our population (Sorato et al. 2018; Zidar et al. 2018a), and can be due to, for example, a seeming lack of a general ‘g’ (Sorato et al. 2018; Qi et al. 2018; but see Shaw and Schmelz 2017).

Variation in dopamine levels can cause optimistic biases (Sharot et al. 2012; Zidar et al. 2018b). To our knowledge, our study is the first to find a link between DRD1 expression and increased optimism. Previously, only D2 receptors have been thought to be associated with optimism, although this has only been seen in the processing of emotional stimuli in humans (Blasi et al. 2009). Our results suggest that D1 receptors, not D2, are involved in avian optimism. On the other hand, we saw that lower 5HT2A expressions tended to be associated with higher optimism. Traditionally, the environment has been thought to influence optimism more than underlying genetics (Harding et al. 2004; Roelofs et al. 2016; Sorato et al. 2018), with particularly enriched environments leading to individuals being more optimistic (e.g., Brydges et al. 2011; Zidar et al. 2018b). Nevertheless, our results indicate a link between gene expression and optimism. In general, increased serotonin is associated with more optimistic biases (Seymour et al. 2012). However, that lowered 5HT2A expression and higher DRD1 expression are linked to higher optimism in our chicks suggests that the role of these monoaminergic systems in avian optimism may be more complex than initially thought, with different receptors having different effects on optimism. More research is, therefore, needed to further explore the role of these monoaminergic systems in optimism.

## Conclusions

Here, we have shown that variation in brain gene expression can be linked to individual variation in cognition, specifically flexibility in reversal learning, and optimism. This confirms the role of the monoaminergic systems in behaviour and and cognition, with the serotonergic receptors, 5HT2A and 5HT2B and dopaminergic receptor, DRD1, appearing to be particularly influential. Future research should examine the details of these relationships, and aim to provide conclusive causal evidence of observed links. Also, the current study included analysis only on the left hemisphere, and thus, comparison between the two hemispheres may reveal further underlying differences with link to phenotypic traits.

## Electronic supplementary material

Below is the link to the electronic supplementary material.

Supplementary file1 (XLSX 22 kb)

Supplementary file2 (PDF 829 kb)
