# Supplementary material for: The relationship between monoaminergic gene expression, learning, and optimism in red junglefowl chicks
Source: Anim Cogn. 2020 May 21;23(5):901–11. doi: 10.1007/s10071-020-01394-z (PMC7415762; doi:10.1007/s10071-020-01394-z)
Supplement: Supplementary file 2 — Supplementary file2 (PDF 829 kb) [file 10071_2020_1394_MOESM2_ESM.pdf]

Supplementary information of Boddington, Gómez Dunlop et al.

'The relationship between monoaminergic gene expression, learning, and optimism in red junglefowl chicks'.

Animal Cognition, https://doi.org/10.1007/s10071-020-01394-z

Robert Boddington[1,2#], Clara A. Gómez Dunlop[1,2#], Laura C. Garnham[1], Sara Ryding[1,2], Robin N. Abbey-Lee[1], Anastasia Kreshchenko[1,2], Hanne Løvlie[1]*

[1]Department of Physics, Chemistry and Biology, IFM Biology, Linköping University,

SE-581 83 Linköping, Sweden.

[2]School of Biological Sciences, University of Manchester, M13 9PL Manchester, UK.

*Corresponding author, email: hanne.lovlie@liu.se

Figure S1. Relationship between reversal learning latency and 5HT2A expression levels in red junglefowl chicks. a) With two extreme gene expression values removed. b) With two extreme gene expression values and four extreme reversal learning latency values removed.

Figure S2. Relationship between reversal learning latency and 5HT2B expression levels in red junglefowl chicks. a) With two extreme gene expression values removed. b) With two extreme gene expression values and four extreme reversal learning latency values removed.

Figure S3. Relationship between optimism and 5HT2A expression levels in red junglefowl chicks, with two extreme gene expression values removed.

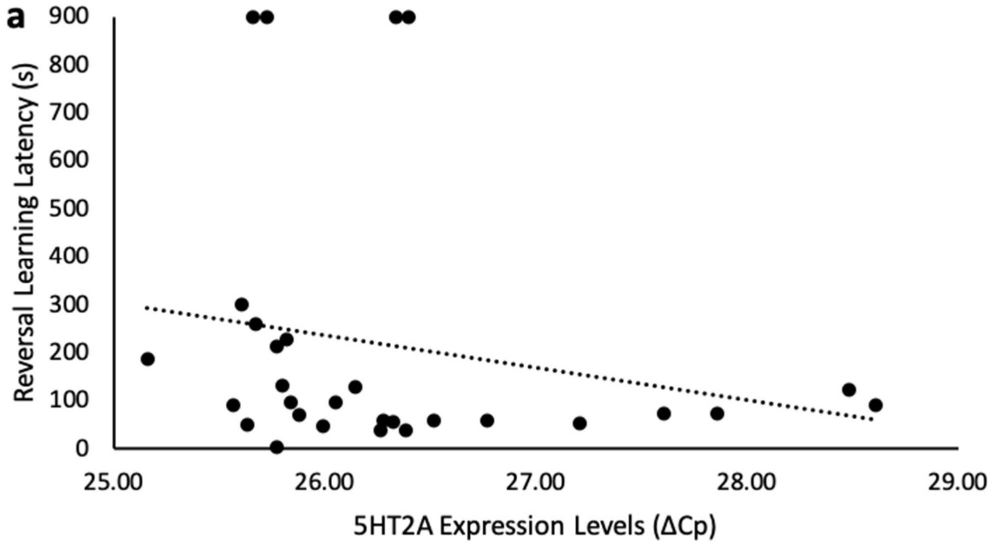

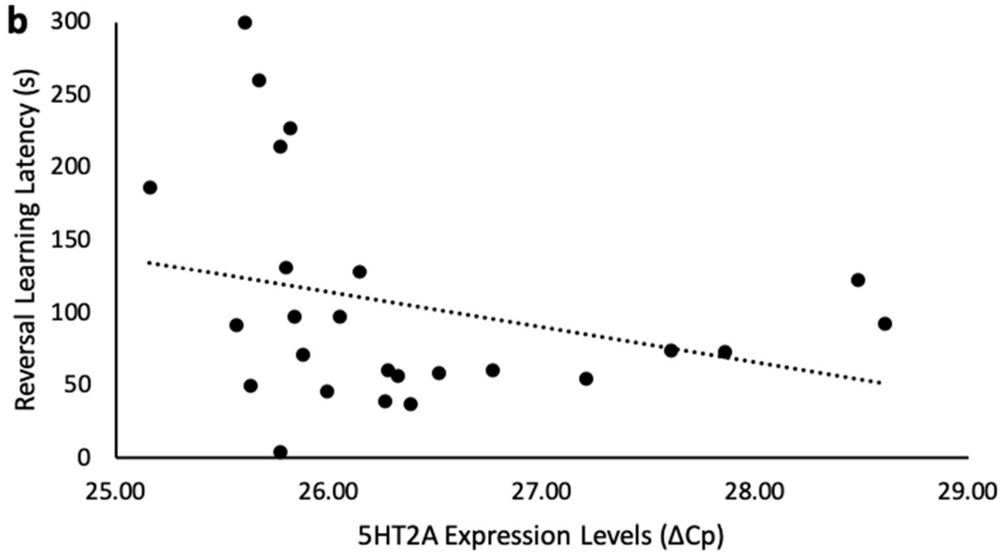

Fig. 1 Relationship between reversal learning latency and 5HT2A gene expression levels in red junglefowl chicks. **a**) With two extreme gene expression values removed (n = 29, $r_s = -0.29$, p = 0.13). **b**) With two extreme gene expression values and four extreme reversal learning latency values removed (n = 25, $r_s = -0.33$, p = 0.11). Reversal learning latency is latency (in seconds) to stop choosing a previously rewarded stimulus in a reversal learning task and instead choose the now rewarded stimulus. A longer latency indicates a less flexible response. Gene expression levels are measured by ΔCp, which is the difference between the gene of interest and a housekeeper gene. Higher ΔCp value indicates lower expression levels.

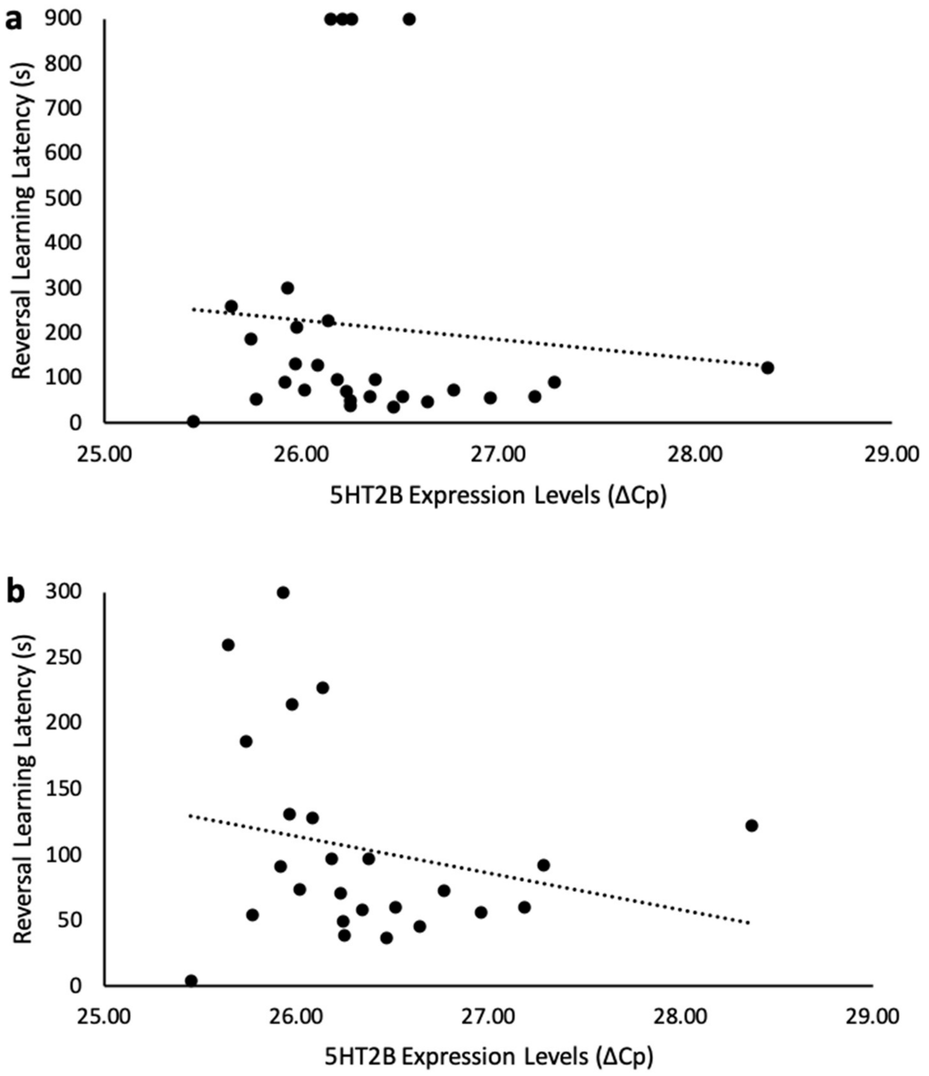

Fig. 2 Relationship between reversal learning latency and 5HT2B gene expression levels in red junglefowl chicks. **a**) With two extreme gene expression values removed ($n = 29$, $r_s = -0.21$, $p = 0.27$). **b**) With two extreme gene expression values and four extreme reversal learning latency values removed ($n = 25$, $r_s = -0.31$, $p = 0.14$). Reversal learning latency is latency (in seconds) to stop choosing a previously rewarded stimulus in a reversal learning task and instead choose the now rewarded stimulus. A longer latency indicates a less flexible response. Gene expression levels are measured by ΔCp, which is the difference between the gene of interest and a housekeeper gene. Higher ΔCp value indicates lower expression levels.

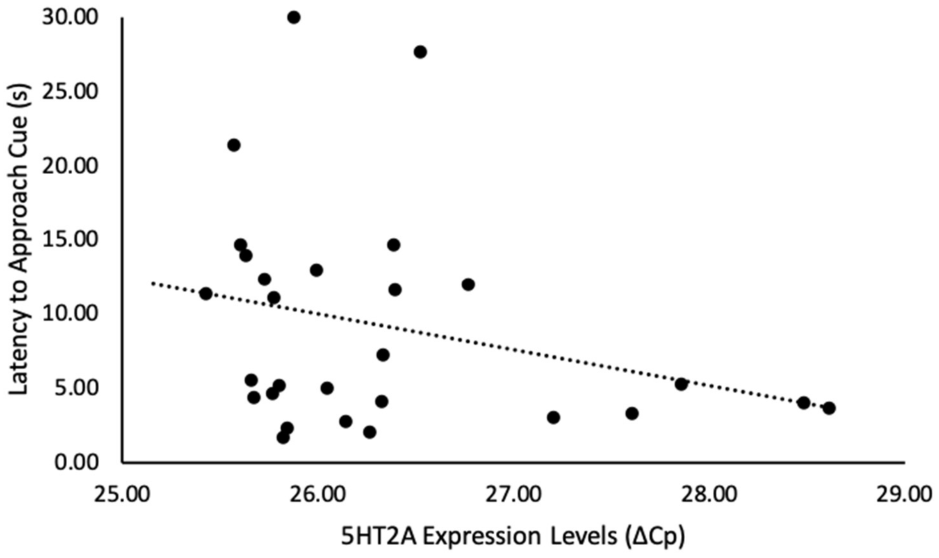

Fig. 3 Relationship between optimism and 5HT2A gene expression levels in red junglefowl chicks (with two extreme gene expression values removed, n = 28, r_s = -0.29, p = 0.13). Latency to approach cue is the used measure of optimism, which is measured as latency (in seconds) to approach a novel, intermediate stimulus in a judgement bias test. A shorter latency indicates a more optimistic response. Gene expression levels are measured by ΔCp, which is the difference between the gene of interest and a housekeeper gene. Higher ΔCp value indicates lower expression levels.
